# Advancing the Integration of Digital Health Technologies in the Drug Development Ecosystem

**DOI:** 10.2196/67052

**Published:** 2025-07-31

**Authors:** Sakshi Sardar, Cheryl D Coon, Scottie Kern, Huong Huynh, Diane Stephenson, Joshua Rubin Abrams, Grace V Lee, Cecile Ollivier, Joseph A Hedrick, Martijn LTM Müller, Luc J W Evers, Lada Leyens, Collin Hovinga, Shu Chin Ma, Klaus Romero

**Affiliations:** 1 Critical Path Institute Tucson, AZ United States; 2 Critical Path Institute Amsterdam The Netherlands; 3 Center of Expertise for Parkinson and Movement Disorders, Department of Neurology, Donders Institute for Brain, Cognition and Behaviour, Radboud University Medical Center Nijmegen The Netherlands; 4 Takeda Zurich Switzerland; 5 Regulatory Science, DEEP Measures Oy Helsinki Finland

**Keywords:** digital health technologies, drug development, DHT, digital health, drugs, disease management, clinical research, technologies, frameworks, viewpoints

## Abstract

Optimized frameworks for efficient and scalable deployment of digital health technologies (DHT) are needed to address existing bottlenecks and unlock the opportunities for remote monitoring and operationalizing decentralized trials. DHTs offer immense potential opportunities for transformation in drug development by providing remote, high frequency, longitudinal insights into physiological processes, and how participants feel and function. Currently, DHT-based drug development tool–related efforts have yielded valuable insights into effective practices and areas that need improvement. However, the development of the required infrastructure is a resource-intensive task, and its efficiency can be greatly enhanced by systematically identifying the required components and aligning them in ways that will avoid trial-and-error approaches by various stakeholders. In this perspective paper, we aim to highlight these crucial aspects required for supporting the rapid and large-scale deployment of DHTs. We propose the development of various standardized consensus frameworks to clearly lay out processes for various stakeholders and facilitate the seamless integration of the next generation of health care–sensing technologies into drug development.

## Introduction

Digital health technologies (DHTs) are integrated in a transformative way into drug development processes [1-3]. The opportunities that exist because of this transformation are substantial, including a longitudinal understanding of how trial participants feel and function while minimizing patient burden. DHTs have the potential to transform the landscape of drug development and health care practice and delivery [4-6]. The rapid advancements in sensor miniaturization and connectivity have led to the widespread use of wearables and Internet of Things, enabling the monitoring of activity, sleep, signs and symptoms of disease, and metabolites among other parameters [7-10]. Many sensing and interactive data collection capabilities have been integrated with the broader infrastructure of health care and drug development to advance the digitalization of acquiring signs and symptoms manifestation and patient experience [9-11]. These sensing technologies can play a crucial role in various phases of drug development and real-world health care settings. Regulatory agencies and the drug development community have well-defined regulatory pathways for drug development tools, including clinical outcome assessments (COAs), biomarkers, and fit-for-purpose model initiatives [12,13]. With the unprecedented pace of technological advancements, streamlined process flows are needed for incorporating new technologies into existing workflows. Globally, regulatory agencies and health care organizations have recognized this potential and have developed programs and guidance to support this transformation [14-16]. To realize a future where health monitoring and assessments with DHTs are effectively integrated into everyday life while maintaining security, privacy, and robustness in decision-making, we must address unresolved challenges associated with the development of verified and validated solutions and their deployment in an ethical manner. Addressing these challenges will aid in accelerating the pace at which new developments in DHT-based solutions can be safely integrated into the larger drug development and health care ecosystem. In this viewpoint, we identify some of the major challenges encountered in the integration of DHT-based solutions in the drug development ecosystem and propose practical recommendations that will require collaborative development.

**Figure 1:**
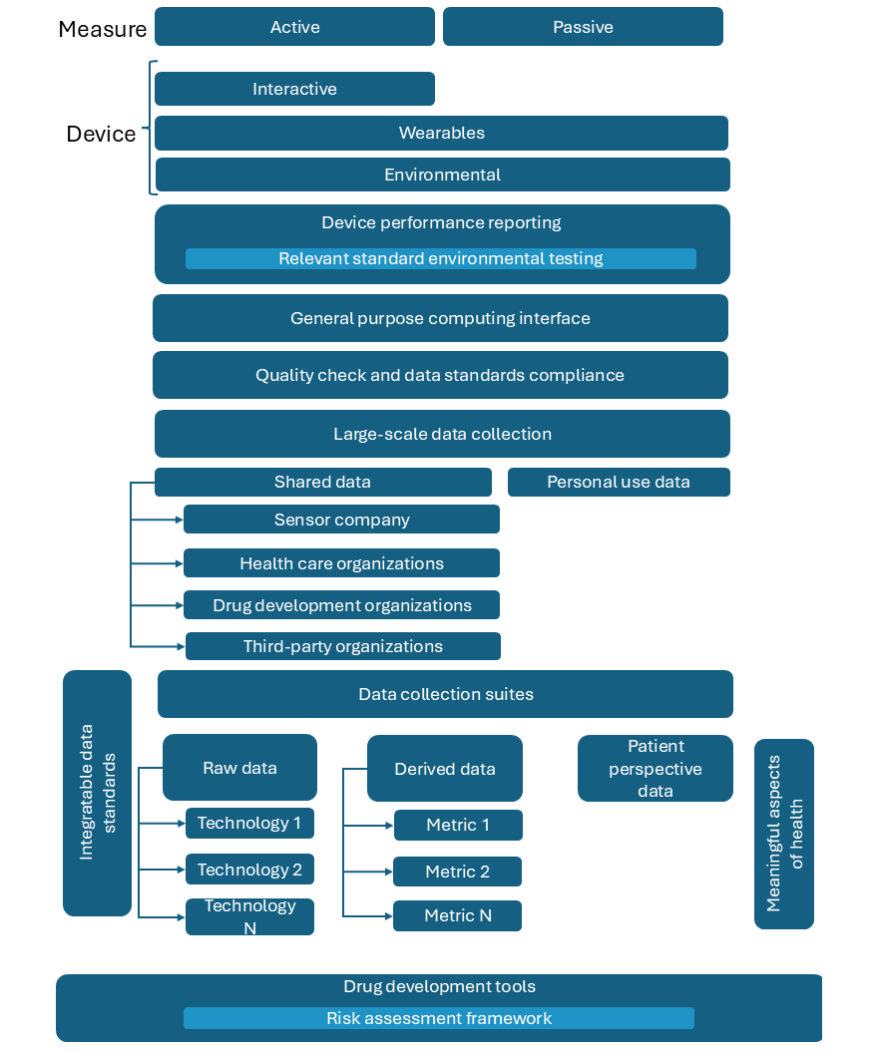
Overview of the needs for consensus-driven frameworks across the process flow for drug development tools. The figure shows the areas where collaborative work is needed to develop standardization and frameworks to support long-term, efficient integration of digital health technologies in the drug development ecosystems. The color scheme difference for certain elements is to highlight the applicability of a subcomponent framework to a major category represented by the bigger rectangle.

The viewpoint is organized by listing challenges in the sequence they are encountered during the data and analysis flows for the DHT-based solution development, as shown in Figure 1. The process starts with the DHT data collected in the form of active or passive measures through wearables, sensors in ambient environments, or interactive technology. The next set of blocks in the figure highlight device-specific needs, such as standardized device performance reporting, standardized evaluations of devices, and standardization to support integration of sensor-based assessments on the general-purpose computing platforms or bring-your-own-devices. Next, we explore blocks representing data collection needs, including quality checks, compliance with the existing data standards, large-scale data collection with various kinds of DHTs, data flow to different stakeholders, and needs around managing relevant data for different contexts. Finally, we focus on the block representing various data analytics requirements to support evidence generation for building DHT-based drug development tools.

## Standards for Reporting DHT Performance Metrics

The reliable utilization of DHT for clinical investigation hinges highly on the verification and validation of the process used to generate the necessary data and its subsequent processing. A major component of this process is DHT-related testing and validation. In many cases, certain components of the DHTs are proprietary and cannot be accessed. The extent of proprietary information varies with the DHTs in question, making it difficult to carry out the necessary verification and validation processes. However, if standards for DHT’s performance for context-of-use (COU) and ground-truth methodologies can be established, DHT evaluations can be streamlined for COUs. Bringing together DHT manufacturers, regulators, and drug developers to reach a consensus on evidence-driven metrics and providing the identified necessary information can drive the development and adoption of standards for reporting on the performance of the DHTs for specific use cases in drug development. This can enhance transparency and facilitate the reliable utilization of DHTs in drug development and clinical practice decision-making. Additionally, these metrics can be included as a part of the framework proposed by Hill et al [17] to provide necessary DHT performance–related contextual information. A component of this concept [18,19] has also been discussed with the European Medicines Agency in support of device-agnostic development, where they have been in favor of setting performance requirements for certain DHT categories associated with a novel end point, enabling other DHT manufacturers to validate against the defined set of performance requirements.

## Standardization of Environmental Factor Assessment for Real-World Application of DHTs

People’s immediate living environment and location can exhibit significant variability, sometimes reaching extremes (eg, size of living space, temperature, altitude, level of internet connectivity, and seasonality). Local environmental factors that can affect the performance of health sensors should be identified and assessed in advance to mitigate any disruptions in downstream analysis arising from them. There is an unmet need for defining the environmental parameters corresponding to the protocols and real-life scenarios for both DHTs and DHT-based assessments. Establishing these categories will streamline the evaluation of DHTs and assessment scenarios. For instance, from the DHT perspective, it is essential to specify deployable geographic locations or operational ranges of environmental parameters, along with the duration for which stable quality output is guaranteed. From the assessment perspective, seasonal, geographic, and occupational variations among other factors can have a direct impact on measurements in a real-world setting. Contextual information related to these factors may aid in interpretability and quantifying uncertainty in the data on the analysis level. The development of frameworks for identifying and standardizing environmental factors and scenarios that should be tested for real-world deployment of DHTs for clinical investigations can immensely support the verification and validation process. The work done by Roussous et al [20] on the identification of various sources of variability can act as a foundation on which the proposed framework can be built. Additionally, exploring the incorporation of risk-based approaches to determine the necessary aspects of environmental factors that must be assessed to inform the extent of validation requirements will be crucial.

## Standardizations to Support Assessments on General-Purpose Computing Platforms or Bring-Your-Own-Devices

The decentralization of trials is expected to increase the utilization of remote sensing technologies [21]. The ways to achieve this involve participants either using DHTs provided by the sponsor or their own. For smaller-scale trials and exploratory purposes, sensor-specific DHTs provided by the sponsors might be suitable. However, this approach becomes problematic as trials scale up, especially when sponsors aim to include a more geographically diverse population or if we use DHTs in pragmatic trials that are embedded in clinical practice or in real-world data studies. Additionally, individuals might be hesitant to wear multiple DHTs measuring different parameters for usability reasons and sometimes because of the stigma it may lead to. As technologies mature, a possible solution can be the integration of these sensors with general-purpose computing platforms like smartphones and wearables. Progressing to this level would depend heavily on the standardization of processes as sensing technologies mature. There is an unmet need for standards that can help implement assessments in a DHT-agnostic manner to truly decentralize future trials and streamline process flows for software updates and new apps using similar functionalities on multipurpose computing systems. Computer system validation frameworks and good practice guidelines need to be reconsidered in this context, as when DHT advances, changes in technology during the study may become inevitable and current validation frameworks might not support this reality. At the same time, regulatory bodies need reliable data to inform their decision-making, it is therefore crucial that reliability and other characteristics can be ensured in the bring-your-own-device (BYOD) framework. Additionally, frameworks for standardizing privacy and consent practices and the interaction of DHT-based clinical assessments with other apps on general-purpose computing platforms such as BYODs are essential.

## Quality Check and Data Standards Compliance Frameworks During Data Collection Processes

The development and implementation of standardized frameworks are needed, not only to minimize or explain missing data but also to support the compliance of collecting standardized data and to perform ad hoc quality checks. These frameworks will help minimize the collection of nonusable data and correct unforeseen issues early in the process of data collection. The proposed quality check and data standards compliance frameworks are different from the standards for data collection. The purpose of these frameworks is to ensure that data are collected in line with the data standard of choice and to perform additional data quality checks associated with data collection to minimize errors. Several platforms exist with built-in quality checks, and various frameworks and recommendations have been proposed to address different component needs [22-25]. However, there remains a lag in achieving consensus on these frameworks and recommendations to attain their appropriate adoption, leading to issues in the data collected in studies.

## Large-Scale Data Generation Management

DHTs allow for unprecedented data generation that will only grow as these technologies advance. As we work toward expanding the deployment of DHTs for clinical investigation, the utilization in natural history and observational studies is expected to rise to support disease characterization. Additionally, DHT-based monitoring will become integral to health care delivery to manage diseases. The decentralized deployment of DHTs will open unprecedented avenues of data collection to gain insights into the health and disease states and develop solutions based on these insights. Raw sensor data from health care–sensing technologies are typically high frequency and high-dimensional, involving different sensor types (eg, accelerometer, gyroscope, photoplethysmography, and electrodermal activity) and different sensor body locations (eg, wrist, ankle, and lower back). Therefore, the decentralized deployment will also bring challenges including large-scale data storage and management to allow for efficient and necessary data flows to appropriate stakeholders. Integrating the aspects of consent for data sharing would be needed to direct the data flow to appropriate stakeholders, for instance, personal users, DHT companies, drug developers, health care organizations, or regulatory agencies. An open question in this field is what constitutes source data for data collected by DHTs: the raw data collected from the patient or the derived variable that is used as the outcome measure, or something in between? Additionally, can derived metrics at the sensor level using validated algorithms be considered source data? This is important as it will determine the extent of data that needs to be stored for regulatory purposes. The drug development and the health care delivery ecosystem must proactively work toward solutions that will efficiently handle these extensive datasets and connectivity between different systems to support regulatory insight generation, record-keeping, processing, and solution development.

## Data Standards Suites

**Figure 2:**
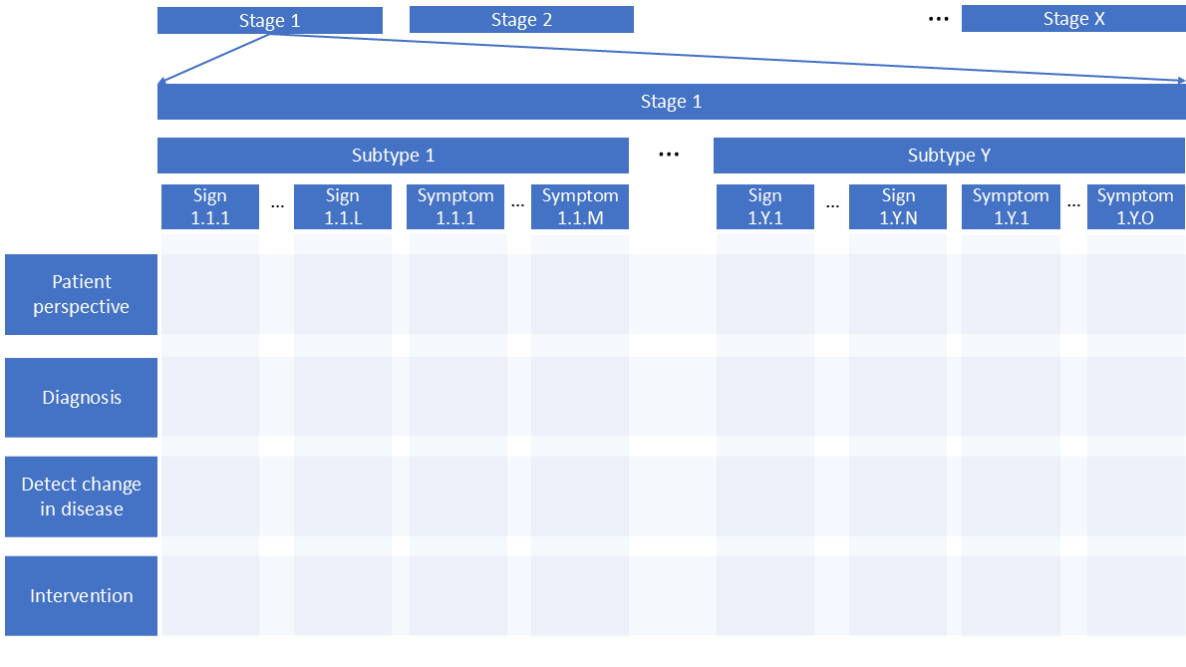
Overview of the areas for categorizing data standards. The figure represents a binning structure that can support data standard suits applicable for data needs related to concepts of interests and contexts of use, including biomarkers, clinical outcomes assessments, and models in various disease areas.

**Figure 3:**
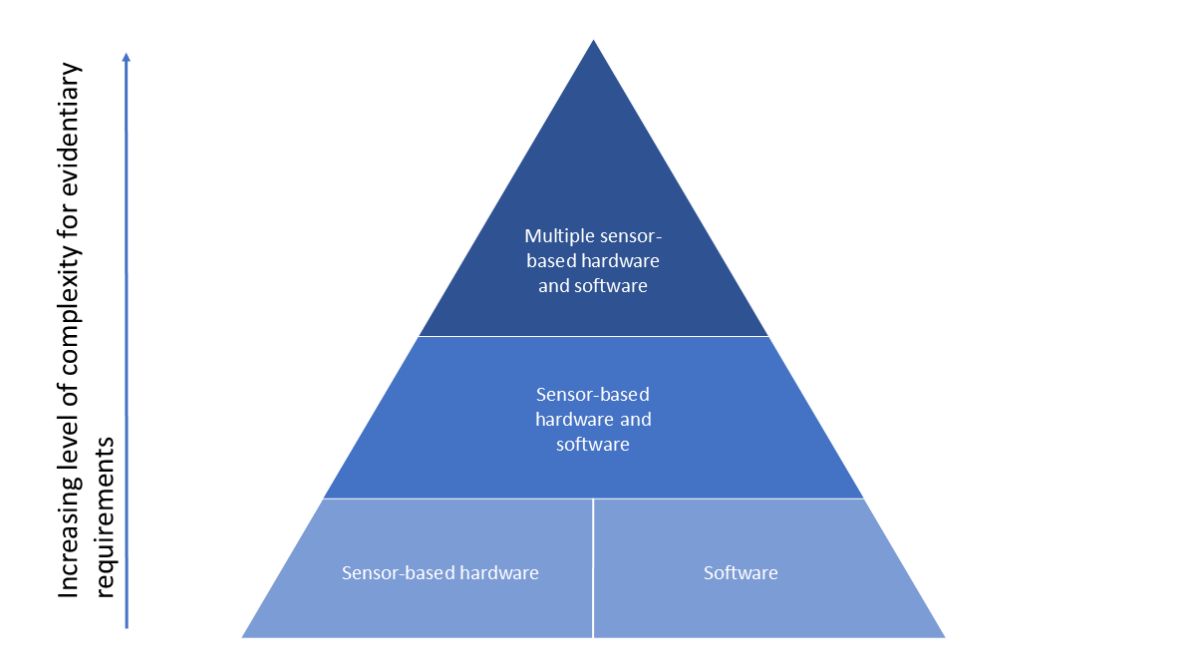
Overview of evidentiary requirement complexity. The figure shows the increasing complexity associated with the evidentiary requirements based on the type of fit-for-purpose sensing technology used for assessing biomarkers, clinical outcomes assessments, or fit-for-purpose models.

To advance the adoption of DHTs in clinical investigations, there is a need for a framework that integrates data standards that account for developments in technological fields. The foundational groundwork for the DHTs has been laid by various initiatives at different organizations like Open mHealth, Clinical Data Interchange Standards Consortium (CDISC), and Critical Path Institute [17,26,27]. However, the lack of consensus standardization and widespread adoption hampers the generation or the reuse of data beyond the primary study objective. Several barriers contribute to this challenge, including (1) an evolving DHT landscape due to technological innovation and the maturation of existing technologies, affecting their performance characteristics; (2) evolving data requirements based on assessment needs; and (3) a lack of standardized catalogs of measurements for the different context of use that can inform the inclusion and exclusion of necessary elements for standardization. Consensus-driven data standards, determined by long-term data standard goals, are needed to overcome these challenges. These long-term goals should be set by stakeholders leveraging collective knowledge and expertise, as no single group or entity has a comprehensive view spanning the entire process flow from study design to its execution across different disease areas. Developing data standard suites that can be a collection of data standards applicable to the concepts of interest and context of use can address the extensive variations in data needs arising due to disease-specific needs. Figure 2 highlights the modular approach to bin the various aspects of data needs relating to concepts of interests and contexts of use that can encompass various biomarkers, clinical outcomes assessments, or models. The modularity provided by each matrix element when combined with the evidentiary requirements listed in Figure 3 can aid in the development of suites of data standards. An approach that can be taken is the establishment of a high-level framework to support bottom-up building up of the requirements based on COUs. The establishment of modular data standard suites can provide an opportunity for stakeholders to select appropriate standards and collect necessary data from various technologies, ensuring minimum requirements for the COU are met.

## Derivable Metrics’ Concept and Its Consistency

In this viewpoint, we define derivable metrics concepts as the metrics that can represent a measurable physical construct that is independent of the algorithmic process or the technology used. For instance, steps are a measurable physical process that can be derived through various sensing technologies like cameras, accelerometers, pressure sensors, and through the use of different algorithms. Metric consistency can become an integral component for supporting innovation and the evolution of newer models, brands, versions, or technologies, and the concept of BYOD construct and solutions created with legacy datasets could benefit greatly from it. We need processes and methodologies for assessing the feasibility and implementation of the derivable metrics concept and its consistency. Such processes can benefit various stakeholders, including patients, DHT companies, drug developers, and regulatory agencies. These will enable the development, utilization, and assessment of the evolving DHTs. Therefore, the development and standardization of the derivable metrics concept and its consistency are essential for long-term success.

## New Methodologies and Processes for Establishing Meaningfulness and Relevance of a DHT-Based Metric

Conventionally, we have relied on patient, caregiver, and clinician observations of signs or symptoms related to disease areas. These observations form the basis for many of the assessments we use today. However, DHTs have enabled the assessment of granular clinical features and processes that were previously inaccessible. For example, with DHTs, we can identify small changes in the symmetry, time duration, and angles associated with walking [28]. We have yet to tap into the full potential of passive monitoring to complement conventional assessments with continuous and objective insights into the course of disease signs in daily life, for example, the frequency and intensity of fluctuating signs and their association with disease states [29]. With such insights, there is an unprecedented need to connect the granular features identified by these DHTs with both clinical relevance and meaningfulness, especially in the context of clinical investigations. It is required to bridge technical implementation with patient-centric outcomes and the lack of this connection can be a major bottleneck for a DHT-based measure and its regulatory approval. In many scenarios, existing methodologies might not be sufficient to bridge the connection between measures and meaningfulness. For instance, certain features based on the frequencies present in a DHT signal might not have interpretable characteristics for the patients, making it challenging to provide information related to their meaningfulness. Certain features are granular, making it difficult for the patients to provide reliable input on how it affects them. This issue can be further exacerbated if methodologies for gaining insights rely on the patient’s ability to recall the experience from the past few days. The involvement of patients and their feedback are essential to derive reliable input about the importance of any measure in this evolving landscape [30-37]. We need collaborative approaches between patients and the DHT developers to augment the existing methodologies that can help in bridging the DHT-derived metrics with meaningfulness.

## Integrable and Multicomponent Data Standards to Support Simultaneous Use of Multiple Digital Health Technologies and Other Data Streams

The development of methodologies for collecting complementary parameters with different DHTs will support a better understanding of health outcomes. Physical activity metric can be a useful construct in itself, but when combined with respiratory function, cardiac function, lifestyle, and medication use, it can provide a much more comprehensive understanding of the interactions of various systems and help in the development of solutions that are informed by holistic input. Another example can be interstitial blood glucose measures that can be affected by activity levels, food intake, and medication use. Medication use solutions developed with glucose readings, activity measures, and food intake could be more holistic in comparison to the ones based on glucose readings. DHTs enable the observation of diverse parameters, providing information about metabolic, physical, and cognitive functions. Additionally, integrating these findings with environmental factors like temperature, humidity, noise, and so on, can generate evidence of the impact of environmental factors on health outcomes. While the observation of a specific parameter can be useful in certain contexts of use, a comprehensive understanding of the measured process requires the development and deployment of technologies to assess different parameters. These observations will become increasingly prevalent and can pave the way for assessing integrated contributions from diseases, drug therapies, lifestyles, environment, and more. We recommend that the community consider these possibilities and collaborate to develop the necessary frameworks for integrating these multiple derivable metrics and raw data streams. These standards would support long-term and forward-looking endeavors. Additionally, in relation to the topic of meaningful metrics, there will be a need for repurposing or augmenting the methodologies to determine the meaningfulness of combined complementary parameters.

## Incorporating New Guidance Into the Existing Regulatory Pathways for the Integration of Technological Advancements

Operationalization and integration of newly developed guidance by regulatory agencies with the existing ones through consensus-driven frameworks will support the overall ecosystem. In a recent draft guidance document on remote health monitoring, the US Food and Drug Administration (FDA) identified 4 different manners of bundling DHTs with an assessment: sensor-based hardware, software, single sensor-based hardware and software, and multiple sensor-based hardware and software [38]. These scenarios vary widely in the extent of their evidentiary requirements for the validation of the fit-for-purpose DHT and the associated measure, showcasing the progression of system complexity. These additional evidentiary support requirements could be added to the core evidentiary support for the drug development tools in increasing order of complexity (Figure 3). Establishing consensus-driven standardized workflows where incremental components of DHTs can be added based on the specific needs of each measurement solution would aid different stakeholders in submissions and evaluations. Best practice evidence frameworks have been proposed [39-41], as well as structured content models for the submission and review of novel end points, collected digitally [18]; the field needs to work on broader adoption of such types of models based on best practices. Additionally, consensus-driven identification of necessary analysis for different intended uses that is methodology agnostic will support developers of these solutions. It can further be augmented through the necessary regulatory interactions by the stakeholders. The recent fast-paced developments in artificial intelligence and machine learning algorithms have also increased their utilization to derive outcomes from raw sensor data. The regulatory agencies are supportive of incorporating these innovations through the development of guidance [42]. Furthermore, the recent advancements toward personalized or individualized treatment development indicate that system complexity is bound to increase [43,44]. Therefore, strategic incorporation of various new guidance with existing regulatory pathways is needed to support the evolving landscape of DHTs that can reduce uncertainties in drug development programs and regulatory evaluations of necessary evidence.

## Frameworks for Risk Assessments

Risk assessment processes are well established for evaluating medical devices intended for premarket approval and 510(k) submissions. However, it is important to note that DHTs used to collect outcome measures and end points do not necessarily qualify as medical devices if they do not have a medical purpose in the trials (this may vary in different regions depending on their medical device regulations). Some DHTs used in clinical investigations may have undergone certain risk assessments as part of the 510(k) or premarket approval submissions in the United States or the Conformité Européenne (CE) marking process in Europe. It would be helpful to have frameworks that can assess the applicability of existing risk assessments and identify additional risk considerations needed for the evaluation or providing evidentiary support for the application of these DHTs in drug trials. It is important to note that while the risk frameworks for drug development tools and medical devices vary (risk to data integrity for the first and risk to patient safety for the latter), there are synergies that can be leveraged across, and the frontiers need to be explored.

## Standardized Terminology

One of the major bottlenecks hampering the effective advancement of DHT-based solutions for decision-making in drug development and, by extension, health care delivery is the lack of standardized terminology for the decision-making tools and processes leveraging DHTs. This issue has been recognized by various groups, resulting in the identification of necessary processes and the development of new terminologies and definitions tailored for specific contexts [45-48]. The field should align on the use and applicability of established terms and concepts such as biomarker or COA, and whether we still need to add digital in front of them (the fact that these measures are collected digitally does not change their identity as biomarkers or COAs, it just specifies the way they are being collected methodologically). Developing consensus-driven definitions that consider the viewpoints of multiple stakeholders will be crucial for driving adoption. A concerted effort is needed to bring the international audience together to reach a consensus on terminologies for this field. While several initiatives have made significant strides in early definitions, it is essential to consolidate, harmonize, and advocate for a consensus terminology adopted by diverse stakeholders to optimize development.

## Verification and Validation of DHT-Based Metric

Verification and validation of DHT-based metrics is a highly resource-intensive endeavor involving collating necessary pieces of evidence spanning multiple areas like clinical validity, device validity, and so on, from representative data sources. This issue is further exacerbated by the fast-paced evolution of the field and the lack of exemplary use cases to guide subsequent developments. We recognize that verification and validation is one of the major bottlenecks, and several groups have been working diligently to tackle it. Several frameworks have been proposed for DHT-based metric development for their use in drug development [39-41,49]. Despite the lack of several exemplary use cases, these frameworks are creative ways of solving the issue at hand by disseminating the current level of understanding for evidence development that the broader community can use. In this viewpoint, we focus on additional areas that must be addressed to support DHT’s integration.

## Frameworks for Ethical Use of DHTs

The large-scale data generation of DHTs also presents significant ethical challenges that must be addressed to ensure their safe and equitable use in both drug development and clinical practice. Lying at the intersection of medicine and digital technology, DHTs must adhere to ethical considerations from both fields simultaneously [50]. European ethical principles dictated by the eHealth Network describe 4 main dimensions of digital health ethics: basing digital health on humanistic values, enabling individuals to manage their digital health and data, making digital health inclusive, and implementing eco-responsible digital health. Addressing these challenges in practice requires researchers and practitioners to be trained in the ethical, legal, and social implications of DHT use, as different trial designs and contexts of use introduce their ethical challenges [50]. For example, allowing individuals to manage their digital health and data can do more harm than good, especially in psychiatric contexts where access to data may increase anxiety [51]. Moreover, the use of DHTs for the detection of adverse events such as falls, cognitive impairments, or drug reactions can improve patient safety but can require intervention from clinicians or caregivers [52]. Defining a protocol for addressing these adverse events and determining the parties responsible for following up are additional ethical concerns of DHTs. To effectively identify and address these challenges in a systematic fashion, consensus frameworks for the ethical implementation of DHTs must be developed and integrated into training for researchers and clinicians using these technologies, especially in sensitive cases such as mental health.

## Synergizing the Use of DHTs in Clinical Care and Drug Development

The lack of synergized use of DHT across the clinical care and drug development continuum creates inefficiency in the development of these tools and their utilization for decision-making. Manifestations of signs and symptoms are generally evaluated in the clinical care setting. The use of DHTs to gain a better understanding of these signs and symptoms will not only help clinical care providers manage the disease but also provide the foundational elements for the discovery of metrics that can be used for drug development purposes. This is important because of several reasons. The characterization of signs and symptoms with DHT is required for determining the validity and relevance of device and metric use in both settings. Additionally, characterizations of signs and symptoms will help in determining whether existing treatment works by effectively managing them and to what extent. This, in turn, provides the foundation for the discovery and development efforts to identify new or better treatments. We need to break these silos to facilitate information flow and make it more efficient across the drug development and clinical care continuum.

## Examples of Successful Multistakeholder Collaborations or Public-Private Partnerships

The Critical Path for Parkinson’s (CPP) consortium consists of multiple stakeholders from around the world who have agreed to collaborate to advance the regulatory maturity of digital health technologies for Parkinson disease clinical trials. CPP’s Digital Drug Development Tools (3DT) initiative successfully acquired and shared WATCH-PD digital device and clinical data with the industry members. Additionally, 3DT has shared regulatory learnings through publications and by providing consensus recommendations for trials using DHTs [20,53]. Active participation of people living with Parkinson disease in CPP and 3DT working groups and their coauthorship on abstracts and publications, including those with FDA, highlights the integration of patient perspectives and voices in the grant’s research efforts [33,34]. Initiatives like the new open-access Library of Digital Measurement Products for Parkinson disease, a joint effort between the Michael J. Fox Foundation, Critical Path Institute’s CPP consortium, and Digital Medicine Society (DiMe), serve as centralized resources to benchmark the state of digital measurement for Parkinson disease, catalyze novel measure development opportunities, and incentivize data sharing [11].

Another example of a public-private partnership between various stakeholders including the pharmaceutical industry, patient advocacy groups, philanthropic organizations, clinical researchers, the National Institutes of Health, and the US FDA is the Critical Path Institute’s Type 1 Diabetes (T1D) consortium. The T1D consortium has received a qualification opinion from the European Medicines Agency on the use of islet autoantibodies as enrichment biomarkers [54], participation in the workshop for outlining the path for using tumor necrosis factor-alpha (TNF-α) inhibitors in pivotal clinical trials in T1D [55], and supported evidence generation for the use of C-peptide as a surrogate end point in clinical trials [56].

## Conclusion

In this viewpoint, we highlight various practical implications and potential future research opportunities for each of the proposed categories for addressing gaps in the integration of DHTs for drug development. In summary, developing frameworks to address device-specific needs will facilitate evidence generation for device use, performance, and integration with general-purpose computing platforms. Developing frameworks for data-specific needs will facilitate data acquisition and flow to different stakeholders while ensuring the quality and quantity meet regulatory requirements. Finally, consensus frameworks on the analysis requirements and methodologies will pave the way to inform the data collection and the verification and validation processes.

Successful future efforts based on the proposed categories will depend on identifying and collaborating with the required stakeholders to address the gaps. DHT-based solution development for drug development is a highly multidisciplinary approach, and without the appropriate representation from required stakeholders, achieving optimal outcomes will be difficult. Additionally, the identification of appropriate upstream and downstream processes and their alignment with the gaps being addressed will be critical for achieving the synergies within the overall process flow of DHT-based drug development solutions.

The utilization of DHTs in health care holds immense potential for advancing drug development. Although current efforts in the field may be fragmented, these are generating important insights through the early development efforts in various disease areas. It is essential to synergize these efforts across several stakeholders. Collaboration across necessary stakeholders representing pharmaceutical companies, sensor technology and DHT companies, clinical care organizations, academia, regulatory agencies, and patient advocacy groups will drive consensus building for the appropriate challenges. Therefore, we propose precompetitive collaborative approaches including building consensus, standardization, and developing frameworks for addressing various challenges identified in this viewpoint, aiming to propel the field forward.

## Abbreviations

3DT: Digital Drug Development Tool
BYOD: bring-your-own-device
CDISC: Clinical Data Interchange Standards Consortium
CE: Conformité Européenne
COA: clinical outcome assessment
COU: context of use
CPP: Critical Path for Parkinson’s
DHT: digital health technology
DiMe: Digital Medicine Society
FDA: US Food and Drug Administration
PMA: premarket approval
T1D: type 1 diabetes
TNF-α: tumor necrosis factor alpha
